# Lymphome cutané primitif anaplasique à grandes cellules se présentant comme un nodule solitaire

**DOI:** 10.11604/pamj.2013.15.51.2809

**Published:** 2013-06-10

**Authors:** Fadwa Tbatou, Badredine Hassam

**Affiliations:** 1 Service de Dermatologie et Vénéréologie, CHU Ibn Sina-Rabat, Maroc; 2 Faculté de Médecine et de Pharmacie de Rabat- Université MohammedV Souissi-Rabat, Maroc

**Keywords:** Lymphome anaplasique, radiothérapie, lymphome cutané, anaplastic lymphoma, radiotherapy, cutaneous lymphoma

## Image en médecine

Le lymphome anaplasique à grandes cellules est une forme de lymphome T cutané se caractérisant par la présence de cellules tumorales anaplasiques, pléomorphes ou immunoblastiques exprimant pour la majorité d'entre elles (plus de 75%) l'antigène CD30. Il se présente sous forme de nodules unique ou multiples localisés ou sous forme de plaques chez le sujet âgé. L'étape initiale de la prise en charge consiste à distinguer un lymphome strictement cutané d'un lymphome systémique secondairement cutané. Le traitement repose sur la surveillance clinique, la radiothérapie, l'exérèse chirurgicale et la chimiothérapie en cas de lymphome disséminé ou de localisations extracutanées. Nous rapportons l'observation d'un patient de 65 ans qui présentait depuis 6 mois un nodule ulcéré et douloureux de l'avant-bras. Cliniquement, il mesurait 2,5cm de diamètre, avait des limites nettes, une surface érythémateuse ulcérée et était fixe par rapport au plan profond. La peau périlésionnelle était souple. On ne retrouvait pas de lésions similaires, d'adénopathies périphériques ni d'hépatosplénomégalie. Nous avons évoqué les diagnostics de leishmaniose cutanée, de carcinome épidermoïde et de mélanome achromique. L'étude histologique de la pièce d'exérèse chirurgicale était en faveur d'un lymphome anaplasique à grandes cellules CD30+. L'échographie ganglionnaire, la TDM thoraco-abdomino-pelvienne et la biopsie ostéo-médullaire n'avaient pas montré de localisations extra-cutanées. Le diagnostic retenu était celui d'un lymphome T cutané à grandes cellules anaplasiques CD30+ stade T1aN0M0. Le traitement complémentaire consistait en une radiothérapie sur le site du nodule permettant une guérison sans récidive à 2,5 ans.

**Figure 1:**
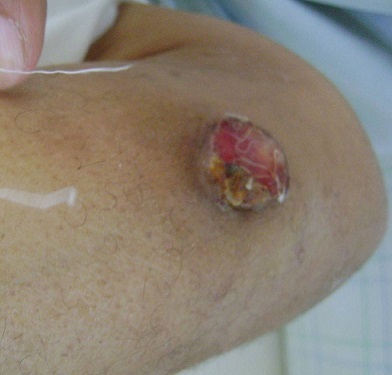
Nodule ulcéré de l'avant-bras

